# Dynamical systems and fractal geometry applied to cardiac dynamics analysis in the Peruvian population

**DOI:** 10.3389/fcvm.2026.1646306

**Published:** 2026-02-05

**Authors:** Sandra C. Correa, Laura D. Ardila, Signed Esperanza Prieto, Jairo Javier Jattin Balcázar, Ribká Soracipa Muñoz, Freddy Andrés Barrios Arroyave, Jose Sulla Torres, Herwin Alayn Huillcen Baca, Herbert del Carpio Beltrán, Giancarlo Christian Alvarez Cervantes, Bárbara Alejandra García Tejada, Joselyn Elizabeth Begazo Paredes, Agueda Muñoz-del-Carpio-Toia

**Affiliations:** 1 Grupo Armonía, Centro de Investigación y Atención Psicosocial Hanami, Bogotá, Colombia; 2 Grupo Insight, Bogotá, Colombia; 3 Vicerrectorado de Investigación, Universidad Católica de Santa María, Arequipa, Perú; 4 Universidad Nacional José María Arguedas, Andahuaylas, Perú; 5 Facultad de Ingeniería y Arquitectura, Escuela Profesional de Ingeniería Industrial, Universidad Continental, Arequipa, Perú; 6 Facultad de Enfermería, Universidad Católica de Santa María, Arequipa, Perú; 7 Hospital Goyeneche, Arequipa, Perú; 8 Hospital Yanahuara, Arequipa, Perú; 9 Vicerrectorado de Investigación, Escuela de Postgrado, Universidad Católica de Santa María, Arequipa, Perú

**Keywords:** cardiac monitoring, heart rate, fractal geometry, dynamical systems, Holter

## Abstract

**Introduction:**

Cardiovascular diseases in Peru are a significant public health problem, and effective methods for risk stratification are needed. Using dynamical systems and fractal geometry shows promising results in other populations.

**Objective:**

To adjust the limits between normality and disease in diagnoses according to the characteristics of the Peruvian population, using a methodology based on dynamical systems and fractal geometry.

**Methodology:**

Heart rate and beats per hour were recorded over 24 h in 272 cases, 193 normal and 79 with arrhythmias, from Holter studies. Spatial occupation of attractors and their fractal dimensions were measured to determine its mathematical state. Results were compared using Cohen's Kappa coefficient with respect to conventional diagnosis. The limits of normality and disease were adjusted to improve concordance with the standard for the Peruvian population.

**Results:**

With the original limits, sensitivity was 0.595, specificity was 0.653, positive predictive value was 0.412, negative predictive value was 0.797, and accuracy was 0.636. The Kappa for the 2 × 2 table was 0.219 (95% CI, 0.104–0.334) and for the 3 × 3 table, it was 0.141 (95% CI, 0.050–0.221). By adjusting the limits, sensibility was 0.430, specificity was 0.839, positive predictive value was 0.523, negative predictive value was 0.783, and accuracy was 0.721. The Kappa for the 2 × 2 table was 0.285 (95% CI, 0.164–0.409) and for the 3 × 3, 0.123 (95% CI, 0.036–0.209).

**Conclusion:**

The agreement of the method improved with the new limits, demonstrating a fair level of alignment, characterized by enhanced specificity but reduced sensitivity. More studies are needed for clinical application.

## Introduction

According to the World Health Organization (WHO), cardiovascular diseases are one of the leading causes of death globally, accounting for over 30% of all recorded deaths worldwide (1). The WHO also asserts that these diseases are preventable, and it is crucial to detect them as early as possible. In different regions of the world, cardiovascular diseases continue to be the primary cause of death (2–6). It is estimated that between 1990 and 2019, the prevalent cases of cardiovascular diseases nearly doubled from 271 million to 523 million, with the number of cases reaching 18.6 million by the latter year (7). More than three-quarters of deaths from cardiovascular diseases occur in low- and middle-income countries, imposing a significant economic burden on these nations (1). In Peru, it is estimated that the loss of gross domestic product (GDP) due to cardiovascular diseases (CVDs) is USD$39.9 billion (8).

According to the Pan American Health Organization, 20% of registered deaths in Peru in 2016 were due to cardiovascular diseases (CVDs) (9), a trend that had been documented since 2010. The mortality rate for cardiovascular diseases in Peru was 73.5 deaths per 100,000 inhabitants in 2019. A significant decrease compared to other countries in the Americas region is highlighted, however, the need to study and analyze the dynamics of this population remains, in order to reduce the percentage of deaths in the country (10).

Estimates of the prevalence of ideal cardiovascular health in Peru show it to be low, as demonstrated by a study that found none of the participants achieved satisfactory cardiovascular health measures (11). Furthermore, the study found that individuals in the lowest socioeconomic tertile, as opposed to those in the middle and high tertiles, were less likely to have ideal cardiovascular health after adjusting the results for sex, age, and education (11). Considering that over 60% of the Peruvian population is overweight (12), it is important to note that even young Peruvians exhibit elevated risk factors for the subsequent development of cardiovascular diseases (13). This situation highlights the high burden and severity of the disease in Peru.

Given this health issue, it is necessary to validate methods that assess the entirety of cardiac dynamics and allow for a quantitative general diagnosis of its condition and severity, which will aid in diagnostic decision-making, distinguishing between severity levels. Clinical diagnosis and physiological investigations fundamentally rely on the capacity to record and interpret complex physiological signals such as heart rate and EEG. Yet, traditional analytical frameworks have lagged behind the technological advances that now enable continuous, long-term, and high-resolution monitoring of these signals (14). These physiological signals have been increasingly recognized as complex, nonlinear, nonstationary, and far-from-equilibrium processes. Nevertheless, many conventional analytical tools still treat them as linear and stationary, focusing primarily on average measures, variability indices, or spectral components. This mismatch between the true dynamics of biological systems and the assumptions of classical analysis often leads to the loss of meaningful information embedded in the intrinsic variability of physiological signals (15). The approach to cardiac dynamics from nonlinear mathematics has allowed for the development of methodologies that distinguish between normality and abnormality, as has been the case in previous studies of Holter recordings, specifically in post-myocardial infarction cases (16), where it has been reaffirmed that the cardiovascular system exhibits nonlinear behavior (17). This means that over time, this type of behavior cannot be expected to adhere to the physical and mathematical principles of a sinusoidal movement. This nonlinearity has been demonstrated in the electrocardiogram (15) in patients at high risk of sudden cardiac death, through an analysis of Heart Rate Variability (HRV). Furthermore, studies have shown a loss of complexity in physiological variability with a reduction in heart rate dynamics before sudden death and aging (16, 18, 19). In addition to dynamical systems, methodologies have been developed to study cardiac dynamics from other theories, such as probability, entropy or fractal geometry (20, 21). No ideas repetidas.

From this perspective, a methodology was developed for evaluating Holter heart rate reports, based on the construction of attractors within the context of dynamic systems theory, and their evaluation using fractal geometry. The results showed that, although fractal dimension is not useful for distinguishing between clinical states, the spaces occupied by attractors in the Box Counting fractal space did allow for differentiation between normal and acute states (21). Subsequent work presented a generalization of the results through the development of an exponential law, establishing quantitative differences between normal and acute states, reporting sensitivity and specificity values of 100% and a kappa coefficient of 1 (22). Additionally, the method provides a stratification of the severity level of any dynamic, establishing an intermediate state termed “chronic”. This methodology looks for a diagnosis applicable to the clinic, which when put into practice would facilitate timely decision-making by the specialist in adverse situations, as well as their proper follow-up.

The importance of this methodology lies in the possibility to establish mathematical orders, whose ranges of numerical values allow for the determination of whether cardiac dynamics are close to or far from an acute state, thus indicating the severity level of the detected alterations (21, 22). However, the complexity of the problem makes achieving this objective quite challenging.

Based on the aforementioned, this research aimed to apply and adjust the boundaries between normality and disease of the described methodology (21, 22) to a sample of the Peruvian population and stablish the concordance between the results and the Holter results, taken as Gold Standard.

## Methodology

### Definitions

**Delay map:** a type of attractor where the dynamics of a system are graphically symbolized, establishing ordered pairs of values of a successive dynamic variable over time in a space of two or more dimensions.

**Fractal:** an irregular object or shape that shows complex patterns repeating at different scales. It has details that remain visible even when magnified and a fractal dimension that is higher than its usual geometric dimension. It also lacks a characteristic scale, meaning its structure cannot be fully described using traditional Euclidean geometry. Instead, fractals are quantified using the fractal (Hausdorff) dimension, which captures their intrinsic geometric complexity. This property makes them especially useful for analyzing irregular physiological signal (23).

**Fractal dimension:** Is a numerical measure used to quantify the degree of irregularity or complexity of an object's geometric structure. In other words, it describes how detail in a pattern changes with the scale at which it is measured. The Box-Counting method will be employed to estimate the fractal dimension. This approach divides the image into grids of progressively smaller squares and counts the number of boxes that contain part of the object's outline. The relationship between the number of occupied boxes and the grid size is then used to calculate the Fractal Dimension.

## Fractal dimension of box-counting

$$D=\frac{LogN(2^{-(K+1)}-LogN(2^{-K})}{Log2^{K+1}-Log2^{K}}=Log_{2}\frac{N(2^{-(K+1)})}{N(2^{-(K)})}$$ (1)

D: Fractal dimension.
N1: Number of squares containing the object's outline with partition grid K.
N2: Number of squares containing the object's outline with partition grid K + 1.
K: Grid partition level 1. K + 1: Grid partition level 2.

The concept of fractal dimension was first introduced by Benoît B. Mandelbrot (24). One of the earliest and most illustrative examples of this concept was the measurement of the coastline of Great Britain, which demonstrated that the measured length increases indefinitely as the measurement scale becomes finer—a phenomenon that inspired the development of the fractal dimension as a mathematical descriptor of natural irregularity (24).

## Procedure

A convenience sampling was conducted, which included a total of 272 Holter reports (which are the summarized versions of the Holter records), with participants over 18 years old without distinction of sex, where 79 belonged to individuals with a history of heart arrhythmias and 193 corresponded to normal individuals, from care health services of a private Cardiology office in the Arequipa Region. It is important to note that the original methodology was applied to cases over 21 years old (21, 22). In this case, 7 cases between 18 and 20 years old were included to determine if there were any mathematical differences in this population.

Initially, the clinical classification of these records was established by a cardiologist specialist. Two types of clinical classifications were performed; the first distinguishes between two states: normality and arrhythmia, while the second establishes a risk stratification into three states: Normality, Critical State, and Intermediate State, which corresponds to all cases not classified in the previous two states. That is, it includes cases with some type of alteration, which can range from normal states with mild alterations to cases diagnosed with arrhythmias that are not severe enough to be considered critical.

**Figure 1:**
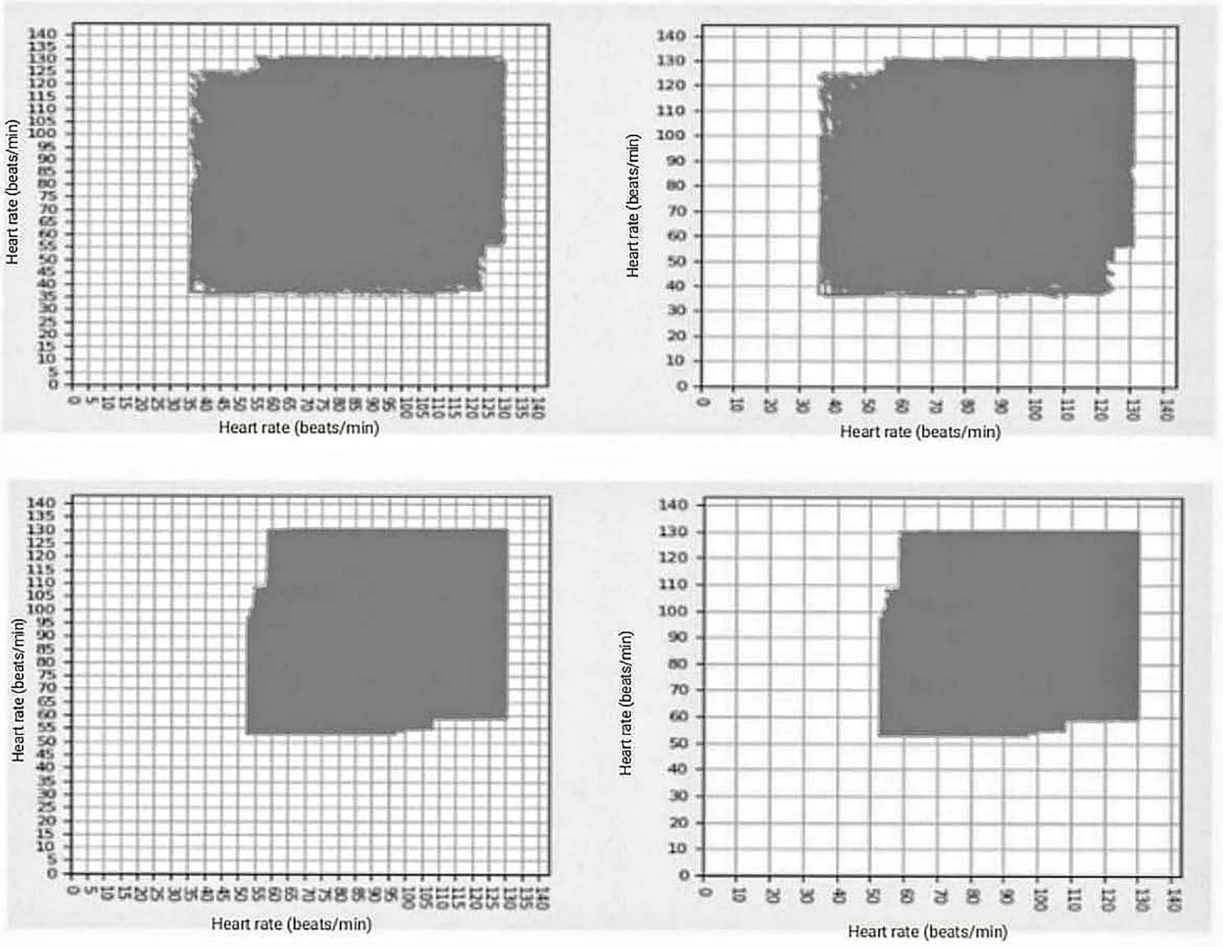
In the upper part are two images corresponding to the arrhythmic patient, with the following values of grids occupied kp = 390, kg = 117, and df = 1.737, in the lower part are the two images corresponding to the grids occupied by normal patient: kp = 277, kp = 80, and df = 1.792.

Next, the maximum and minimum heart rate values were systematized every hour, as well as the total number of beats per hour from the Holter records over 24 h. These values were then used to construct a simulation of the complete sequence of heart rate frequencies, which was subsequently plotted in a delay map to build each attractor. To design the delay map and construct each attractor, a sequence of heart rate values was generated using a random algorithm based on the maximum and minimum heart rate per hour and the total number of beats per hour extracted from the Holter reports (which are the summarized versions of the Holter records). These sequences were then organized in an Excel file and uploaded to the analysis software used for processing. This resulted in a numerical sequence of heart rates, with which ordered pairs were established and plotted on an attractor. Subsequently, the fractal dimension of the attractor was calculated, and the occupation space was counted by overlapping two grids of 5 and 10 beats/min, denoted as Kp and Kg respectively. An example of these attractors for an arrhythmic and a normal patient is shown in Figure 1, illustrating the occupied grids and corresponding values of Kp, Kg, and Df.

**Figure 2:**
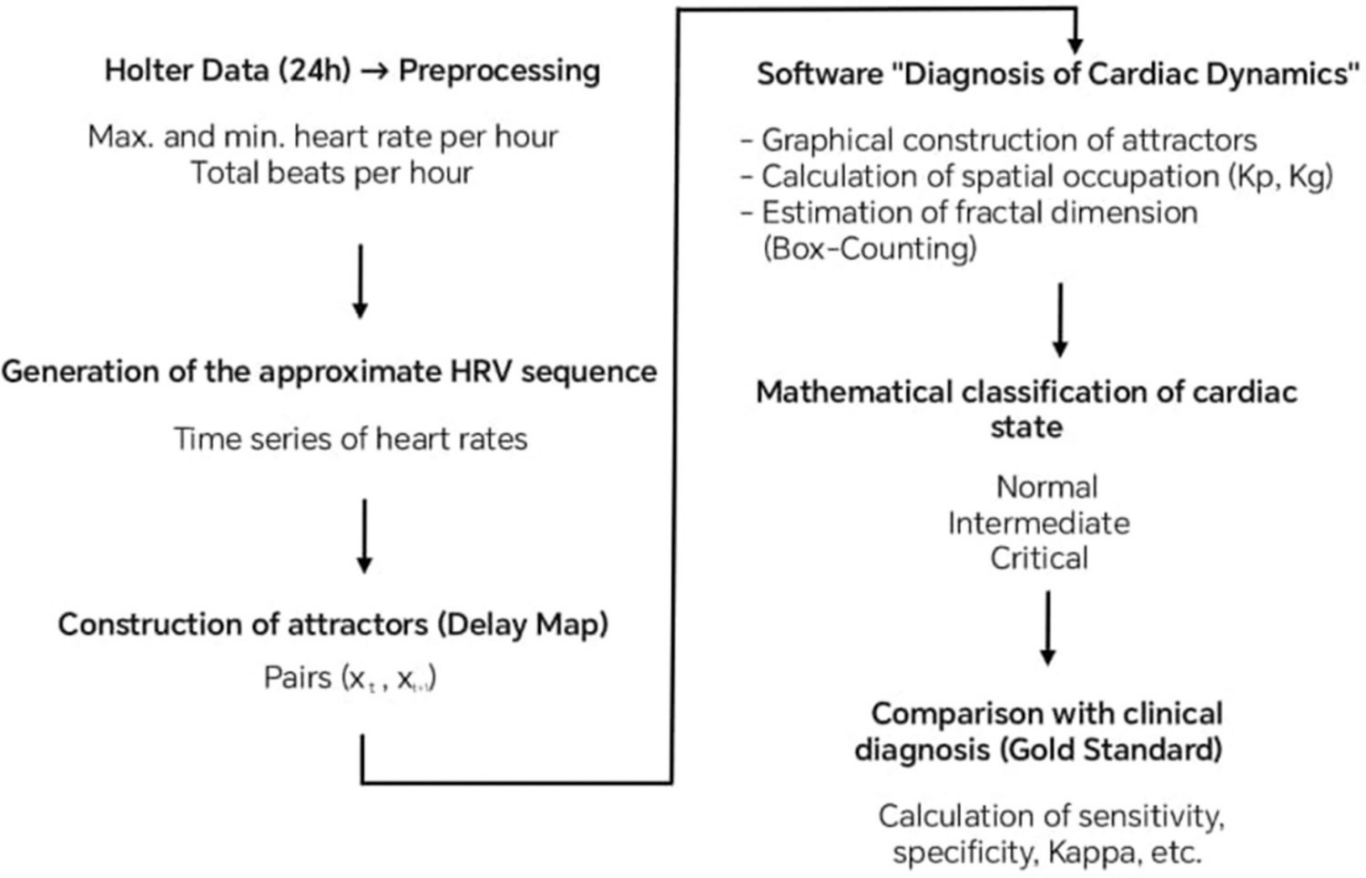
Summary of the study methodology.

Based on these measures and the differentiation criteria established in previous works, the mathematical state of the Holter recordings was classified into three categories: Normality, Critical state, and Intermediate state. To establish it, the occupation spaces of the attractors on the Kp grid were evaluated. Previous studies had shown that the Critical states were associated with values below 100, while Normal cases and some chronic diseases presented values above 200, and values between 100 and 200 corresponded to an Intermediate state or evolution between these states (22). These values were applied to establish the mathematical state of each cardiac dynamic. The entire analytical workflow, from data preprocessing to mathematical classification and comparison with the clinical diagnosis, is summarized in Figure 2.

Following the methodological sequence described above, an additional analytical stage was implemented to assess the discriminative capacity and calibration of the model. For this purpose, receiver operating characteristic (ROC) curves were constructed, and the area under the curve (AUC) with its 95% confidence intervals was estimated using the DeLong method. Considering the imbalance between normal and arrhythmic cases, the precision–recall (PR) curve and the area under the PR curve (AUPRC) were also included, with 95% confidence intervals obtained through bootstrap resampling (2,000 iterations). In addition, model calibration was examined by calculating the Brier score and plotting the calibration curve, which compares the observed vs. predicted probabilities.

To ensure comprehensive performance evaluation, results were analyzed at four decision thresholds: (1) the original threshold, (2) the adjusted threshold, (3) the optimal threshold according to the Youden index, and (4) a clinical threshold focused on achieving a sensitivity of at least 0.85. For each of these thresholds, the main diagnostic metrics—sensitivity, specificity, positive predictive value (PPV), and negative predictive value (NPV)—were reported with their corresponding exact 95% confidence intervals calculated via the Clopper–Pearson method.

Finally, to strengthen the clinical interpretation of the results, the absolute number of false negatives and false positives expected per 1,000 screenings was estimated, taking into account the observed prevalence in the analyzed sample. The stability of model performance under class imbalance was also explored by applying weighting techniques and, where applicable, oversampling using the SMOTE algorithm in scenarios involving multivariate predictors.

## Used application

The construction of attractors and the calculation of mathematical parameters, such as spatial occupation in the Kp and Kg grids and the fractal dimension using the Box-Counting method, were performed using the My Cardio mobile application. This software was developed as part of the project “Cardiac diagnosis and monitoring using a physical-mathematical methodology based on fractal geometry and dynamical systems incorporated in wearable technologies” (Applied Research Projects 2022-2, code PE501080050-2022—PROCIENCIA CONCYTEC). For further details, see Torres et al. (2025) (25).

Originally created for Android-based smartwatches, My Cardio integrates modules for signal acquisition, data storage, and mathematical analysis based on the principles of dynamical systems and fractal geometry. A complementary module was later implemented to analyze Holter summaries derived from hourly maximum and minimum heart-rate data and the total number of beats extracted from Excel files, allowing retrospective simulations of the cardiac dynamic when continuous raw data were unavailable.

## Statistical analysis

After applying the method and obtaining mathematical diagnoses according to the original limits of normality and disease, a comparison of the obtained diagnosis with respect to the clinical standard, taken in this research as the gold standard, was performed. This allowed for the validation of Sensitivity, Accuracy, Specificity, Positive Predictive Value (PPV), and Negative Predictive Value (NPV) of the mathematical method, as well as the Kappa coefficient to assess diagnostic agreement beyond chance. Considering that the objective was not only to establish the method's ability to differentiate between normality and arrhythmia but also its ability to stratify the severity level of pathological states, two Kappa coefficient analyses were performed.

The first analysis established the method's ability to differentiate between Normality and Arrhythmia, for which the Intermediate and Critical mathematical states were combined, that is, both were considered as Arrhythmia, and Cohen's Kappa coefficient was applied through a binary classification using a 2 × 2 table, where:

True positives (TP): Holter with a clinical diagnosis of Arrhythmia and a mathematical evaluation of a Critical or Intermediate state.
False positives (FP): Holter with a clinical diagnosis within normal limits but mathematically diagnosed with Critical disease or an Intermediate state.
False negatives (FN): Holter with a clinical diagnosis of arrhythmia but a mathematical evaluation of normality.
True negatives (TN): Holter with a clinical diagnosis within normal limits and mathematically diagnosed as Normal.

For the second analysis, Cohen's Kappa coefficient adjusted to a 3 × 3 table was applied to establish the ability to stratify pathological cases. For this, the clinical stratification into three states (Normal, Intermediate, and Critical) was compared with the corresponding mathematical evaluation of three states.

Then, to assess diagnostic performance in detail, the discriminatory power of the mathematical index (Kp) was evaluated using receiver operating characteristic (ROC) curves and the area under the curve (AUC), with Kp treated as a continuous predictor (score = −Kp, as lower Kp values indicate a higher probability of alteration). Ninety-five percent confidence intervals (95% CI) for the AUC were estimated using bootstrap resampling with 1,000 replicates. For binary classifications (original and adjusted thresholds), sensitivity, specificity, positive predictive value (PPV), negative predictive value (NPV), and accuracy were estimated, together with their exact 95% CIs obtained via the Clopper–Pearson method. In addition, Cohen's coefficient of concordance (Kappa) and its 95% CI were calculated using bootstrap resampling (1,000 replicates). The adjusted threshold was determined by maximizing the sum of sensitivity and specificity (Youden index) within the sample. Both the original and adjusted thresholds are reported for transparency.

## Adjustment of the mathematical limits of normality and disease

As mentioned earlier, it was originally established that spatial occupation values below 100 corresponded to acute disease, while values above 200 corresponded to normal or chronic disease cases (22), with the range between 100 and 200 corresponding to an intermediate state between both conditions. Considering all the mathematical measurements performed, an analysis was conducted to adjust the limits of normality, intermediate state, and disease to the characteristics of the Peruvian population, according to the evaluated sample, thus establishing new values for the mathematical evaluation of each patient.

Subsequently, diagnostic concordance measures were taken again according to the new limits, with the aim of evaluating the improvement in diagnostic capacity. Finally, a comparison between both analyses (the previous and the current one) was carried out to determine performance according to the evaluated concordance measures.

## Justification for the threshold adjustment

The adjustment of the cutoff points was made based on empirical criteria that sought the best match with the characteristics observed within the analyzed sample. The performance of different cutoff values was explored, and those that provided a greater balance and overall agreement with the clinical diagnosis were selected. This approach is based on the fact that the inherent variability of cardiac dynamics may differ between populations, and therefore, strictly applying the limits reported in previous studies could lead to systematic errors in classification. Likewise, this work explicitly assumes that the adjusted values constitute a preliminary approximation and that their clinical validity must be confirmed in further studies with larger and more diverse samples.

## Ethical aspects

The Institutional Ethics Committee of the Universidad Católica de Santa María has reviewed and approved this research, ensuring compliance with national and institutional ethical standards. In accordance with the ethical standards outlined in the *Código de Ética para la Investigación y la Integridad Científica* (CE-VRINV-001/V03) of the Universidad Católica de Santa María, this study adheres to the principles of beneficence, non-maleficence, justice, and autonomy. The research has been classified as minimal risk, as it involves the analysis of non-invasive medical data from clinically prescribed examinations.The study ensures the protection of participant confidentiality and privacy, as mandated by the Resolución Ministerial N° 233-2020-MINSA regarding ethical considerations for research involving human subjects. All sensitive data has been anonymized to safeguard participant identity.

Furthermore, the informed consent process was conducted in alignment with Article 10 of the UCSM Code of Ethics, guaranteeing that all participants provided voluntary and informed consent before participating in the study.

## Results

The studied sample was found to have a higher percentage of women (53%) than men (47%). Their ages ranged from 18 to 95 years, with an average age of 50.3 ± 44.7 years. According to the first classification performed by the expert cardiologist, 193 subjects were classified as Normal, and 79 presented Arrhythmias. Additionally, with the three-level risk stratification, 53 were found to be in a Critical condition, 146 were classified as normal, and 73 subjects were in an intermediate or evolving state between the two previous conditions. An example of the clinical diagnosis of the Holter records can be seen in Table 1.

**Table 1:** Clinical information of 22 of the analyzed cases.

No.	Sex	Age	Holter Report
1	M	53	Baseline sinus rhythm. Self-limited episodes of daytime sinus tachycardia. Isolated supraventricular extrasystoles. No bradyarrhythmias. No evidence of silent ischemia. No significant pauses.
2	F	25	Baseline sinus rhythm. Maximum heart rate of 122 bpm in sinus rhythm. Minimum heart rate of 52 bpm in sinus rhythm. Presence of occasional ventricular extrasystoles. No episodes of ventricular tachycardia. No atrial fibrillation. QT not prolonged.
3	F	34	Symptoms described as “fetal hiccup” not corresponding to arrhythmic disturbances. Average heart rate was 91 bpm during the day, 78 bpm at night, and 87 bpm over the entire recording period. Maximum heart rate was 162 bpm, indicative of sinus tachycardia. Minimum heart rate was 50 bpm. The standard deviation for normal beats was 123.0. A total of 124,703 beats were recorded, of which 9 were classified as ventricular beats, isolated ventricular extrasystoles with no couples or episodes of sustained or nonsustained ventricular tachycardia. Additionally, 5 isolated supraventricular extrasystoles were observed, with no couples or episodes of sustained or nonsustained supraventricular tachycardia.
			Heart rate within normal ranges, preserved circadian rhythm, normal AV conduction, and no significant pauses.
4	F	34	Average heart rate was 90 bpm during the day, 67 bpm at night, and 82 bpm over the entire recording period. Maximum heart rate was 156 bpm. Minimum heart rate was 49 bpm. The standard deviation for normal beats was 142.7. A total of 117,791 beats were recorded, of which 1 was classified as a ventricular beat, isolated ventricular extrasystole with no couples or episodes of sustained or nonsustained ventricular tachycardia. Additionally, 10 isolated supraventricular extrasystoles were observed, one supraventricular couple, with no episodes of sustained or nonsustained supraventricular tachycardia. Heart rate within normal ranges, no significant pauses, normal AV conduction, preserved circadian rhythm.
5	F	25	The record shows sinus rhythm with narrow QRS complexes. Average heart rate of 85 bpm, minimum of 49, and maximum of 148 bpm, maintaining the circadian cycle. Two supraventricular extrasystoles were observed. All episodes recorded as tachycardias are sinusoidal. No pauses, blocks, or complex arrhythmias were observed. The clinical event diary records two episodes of chest pain and dyspnea that do not correspond to specific findings on the electrocardiogram.
6	F	41	The record shows sinus rhythm with narrow QRS complexes. Average heart rate of 83 bpm, minimum of 49, and maximum of 156 bpm, maintaining circadian rhythm. No complex arrhythmias, pauses, or blocks were observed.
7	M	54	No symptoms described. Average heart rate was 91 bpm during the day, 71 bpm at night, and 84 bpm over the entire recording period. Maximum heart rate was 143 bpm. Minimum heart rate was 51 bpm. The standard deviation for normal beats was 135.2. A total of 120,821 beats were recorded, of which 0 were classified as ventricular beats, indicating no isolated ventricular extrasystole, couples, or episodes of sustained or nonsustained ventricular tachycardia.
			Additionally, 12 isolated supraventricular extrasystoles were observed, with no couples or episodes of sustained or nonsustained supraventricular tachycardia. Heart rate within normal ranges, normal AV conduction, preserved circadian rhythm, and no significant pauses.
8	F	39	Study conducted without bradycardic or antiarrhythmic treatment during hospital admission. Sinus rhythm was observed throughout the study. No episodes of atrial fibrillation. No pauses >2.5 s. Average heart rate was 69 bpm during the day, 62 bpm at night, and 67 bpm over the entire recording period.
			Maximum heart rate was 104 bpm at 20:56:57, indicative of sinus tachycardia. Minimum heart rate was 49 bpm during sinus bradycardia while sleeping.
			Attenuated circadian rhythm of heart rate was observed. Normal heart rate variability with low-risk indicators. Predominance of parasympathetic tone was observed. No AV block episodes were recorded. No ST-segment abnormalities were observed. QTc interval within normal ranges. Minimal irrelevant supraventricular extrasystoles (01). No episodes of supraventricular tachycardia. Minimal irrelevant ventricular extrasystoles (01). No episodes of ventricular tachycardia. Some episodes of sleep apnea/hypopnea were observed in the EDR analysis. The patient reported no symptoms throughout the study. Summary: Attenuated circadian rhythm of heart rate. Some episodes of sleep apnea/hypopnea were recorded in the EDR analysis (suggesting polysomnography). The rest is normal (no significant arrhythmic findings).
9	F	57	Sinus rhythm was observed throughout the study. Isolated supraventricular and ventricular extrasystoles were recorded. One episode of sinus tachycardia with a maximum heart rate of 103 bpm was observed. No bradyarrhythmias were observed. No significant pauses were recorded.
10	M	62	No symptoms described. Average heart rate was 79 bpm during the day, 74 bpm at night, and 77 bpm over the entire recording period. Maximum heart rate was 102 bpm. Minimum heart rate was 60 bpm. The standard deviation for normal beats was 59.6. A total of 111,031 beats were recorded, of which 0 were classified as ventricular beats, indicating no isolated ventricular extrasystole, couples, or episodes of sustained or nonsustained ventricular tachycardia. Up to 9 isolated supraventricular extrasystoles were observed, with no couples or episodes of sustained or nonsustained supraventricular tachycardia. Normal AV conduction, no significant pauses, preserved circadian rhythm, and normal heart rate.
11	F	88	Holter study with abundant artifacts. Baseline sinus rhythm with preserved circadian variations in heart rate. No QT or ischemic load abnormalities were observed. Low-density ventricular and supraventricular extrasystoles. Episode of sinus tachycardia up to 134 bpm followed by an episode of atrial fibrillation with elevated ventricular response lasting approximately 6 s, without associated symptoms. No significant pauses were recorded. Conclusion: Asymptomatic paroxysmal atrial fibrillation.
12	F	21	Holter study with abundant artifacts. Baseline sinus rhythm with preserved circadian variations in heart rate. No QT or ischemic load abnormalities were observed. Low-density ventricular and supraventricular extrasystoles. Episode of sinus tachycardia up to 134 bpm followed by an episode of atrial fibrillation with elevated ventricular response lasting approximately 6 s, without associated symptoms. No significant pauses were recorded. Conclusion: Asymptomatic paroxysmal atrial fibrillation.
13	M	76	No symptoms described. Study in sinus rhythm with successive episodes of paroxysmal atrial fibrillation lasting up to 30 s, episodes of sinus tachycardia, and low-density ventricular and supraventricular extrasystoles. No ST-segment abnormalities. No significant pauses were recorded.
14	F	71	Sinus rhythm. No tachycardias or bradycardias. Average heart rate of 87 bpm during the day and 70 bpm at night. Maximum heart rate was 119 bpm and minimum 51 bpm. Three isolated supraventricular extrasystoles and two isolated ventricular extrasystoles were recorded. No pauses or ischemic abnormalities were observed.
15	M	65	Baseline sinus rhythm with an average heart rate of 77 bpm. A nonsustained ventricular tachycardia episode of 9 beats at 178 bpm was recorded. Ventricular and supraventricular extrasystoles were observed. No ischemic abnormalities were observed.
16	M	71	Study conducted without bradycardic or antiarrhythmic treatment. Sinus rhythm throughout the study. No episodes of atrial fibrillation. Average heart rate was 60 bpm during the day, 49 bpm at night, and 56 bpm over the entire recording period. Slightly attenuated circadian rhythm of heart rate (CI: 1.23). Maximum heart rate was 99 bpm, corresponding to sinus rhythm. Minimum heart rate was 41 bpm, corresponding to nocturnal sinus bradycardia during sleep (physiological). No pauses >2.5 s were recorded. No ST-segment abnormalities were recorded. QTc interval within the normal range throughout the study. Infrequent and paucisymptomatic supraventricular extrasystoles (55 recorded), mostly isolated, 5 supraventricular
17	M	63	Estudio realizado en ritmo sinusal. Frecuencia cardiaca media de 82 lpm. La frecuencia cardiaca máxima fue de 108 lpm y la mínima de 63 lpm. Se observaron extrasístoles ventriculares y supraventriculares ocasionales. No se observaron pausas significativas ni anormalidades isquémicas.
18	M	50	Sinus rhythm. Average heart rate of 79 bpm, maximum of 121 bpm, and minimum of 60 bpm. No episodes of tachycardia or bradycardia were observed. Some ventricular and supraventricular extrasystoles were noted. No significant pauses or episodes of atrial fibrillation were observed.
19	F	72	Sinus rhythm with preserved circadian variability. Average heart rate of 67 bpm, maximum of 97 bpm, and minimum of 52 bpm. Some isolated supraventricular extrasystoles and a couple of ventricular extrasystoles were observed. No significant pauses or ST segment abnormalities were recorded.
20	F	68	Sinus rhythm. Average heart rate of 72 bpm, with a maximum of 115 bpm and a minimum of 58 bpm. No tachycardias, bradycardias, or complex arrhythmias were observed. Occasional ventricular and supraventricular extrasystoles. No significant pauses or signs of ischemia were recorded.
21	M	35	An ambulatory Holter recording was initiated on 02/13/2008 at 22:00:23 with a duration of 23:59:59 h. No symptoms were reported. The recording showed sinus rhythm. The average heart rate was 79 bpm during the day, 79 bpm at night, and 79 bpm throughout the entire recording. The maximum heart rate was 121 bpm at 20:55:25. The minimum heart rate was 58 bpm at 19:08:53. The standard deviation of normal beats was 75.6. A total of 113,927 beats were recorded, of which 0 were classified as ventricular beats, with no couplets or runs of sustained or nonsustained ventricular tachycardia. A single isolated supraventricular extrasystole was observed, with no couplets or runs of sustained or nonsustained supraventricular tachycardia. Heart rate was within normal ranges, AV conduction was normal, circadian rhythm was preserved, and no significant pauses were noted.
21	M	35	An ambulatory Holter recording was initiated on 02/13/2008 at 22:00:23 with a duration of 23:59:59 h. No symptoms were reported. The recording showed sinus rhythm. The average heart rate was 79 bpm during the day, 79 bpm at night, and 79 bpm throughout the entire recording. The maximum heart rate was 121 bpm at 20:55:25. The minimum heart rate was 58 bpm at 19:08:53. The standard deviation of normal beats was 75.6. A total of 113,927 beats were recorded, of which 0 were classified as ventricular beats, with no couplets or runs of sustained or nonsustained ventricular tachycardia. A single isolated supraventricular extrasystole was observed, with no couplets or runs of sustained or nonsustained supraventricular tachycardia. Heart rate was within normal ranges, AV conduction was normal, circadian rhythm was preserved, and no significant pauses were noted.
21	M	35	An ambulatory Holter recording was initiated on 02/13/2008 at 22:00:23 with a duration of 23:59:59 h. No symptoms were reported. The recording showed sinus rhythm. The average heart rate was 79 bpm during the day, 79 bpm at night, and 79 bpm throughout the entire recording. The maximum heart rate was 121 bpm at 20:55:25. The minimum heart rate was 58 bpm at 19:08:53. The standard deviation of normal beats was 75.6. A total of 113,927 beats were recorded, of which 0 were classified as ventricular beats, with no couplets or runs of sustained or nonsustained ventricular tachycardia. A single isolated supraventricular extrasystole was observed, with no couplets or runs of sustained or nonsustained supraventricular tachycardia. Heart rate was within normal ranges, AV conduction was normal, circadian rhythm was preserved, and no significant pauses were noted.
21	M	35	An ambulatory Holter recording was initiated on 02/13/2008 at 22:00:23 with a duration of 23:59:59 h. No symptoms were reported. The recording showed sinus rhythm. The average heart rate was 79 bpm during the day, 79 bpm at night, and 79 bpm throughout the entire recording. The maximum heart rate was 121 bpm at 20:55:25. The minimum heart rate was 58 bpm at 19:08:53. The standard deviation of normal beats was 75.6. A total of 113,927 beats were recorded, of which 0 were classified as ventricular beats, with no couplets or runs of sustained or nonsustained ventricular tachycardia. A single isolated supraventricular extrasystole was observed, with no couplets or runs of sustained or nonsustained supraventricular tachycardia. Heart rate was within normal ranges, AV conduction was normal, circadian rhythm was preserved, and no significant pauses were noted.
22	M	90	Sinus rhythm as baseline with narrow qrs, average heart rate of 80 bpm, maximum heart rate of 113 bpm at 11:26, minimum heart rate of 54 bpm at 09:41, very low-density caps, no ventricular arrhythmia, adequate heart rate variability, no significant pauses.

The fractal dimension of normal and arrhythmic cases ranged from 1.39 to 2. These results corroborate previous findings that indicate fractal dimension values do not allow distinctions between cardiac dynamics, as the value ranges for both states overlap (26).

The attractors of clinically normal cases evaluated with the Kp grid showed occupation spaces that varied from 47 to 1,109, while for arrhythmic dynamics, the range was between 18 and 1,572. On the other hand, the spatial occupation of attractors in the Kg grid ranged between 15 and 290 for normal cases and between 7 and 583 for arrhythmic cases (see Table 2).

**Table 2:** Mathematical measures, comparison between clinical diagnosis and mathematical diagnosis of 22 evaluated cases. Clinical Status: binary differentiation between normal and Arrhythmia. Clinical Stratification: clinical classification into three states. Kg: Spaces occupied by the attractor in the 10 beats/minute grid. Kp: Spaces occupied by the attractor in the 5 beats/minute grid. F: fractal dimension. Original Mathematical Stratification: mathematical classification with original limits. Adjusted Mathematical Stratification: classification with limits adjusted to the Peruvian population sample.

No.	Clinical Status	Clinical Stratification	Kp	Kg	DF	Original Mathematical Stratification	Adjusted Mathematical Stratification
1	Normal	Normal	1,109	290	1.93	Normal	Normal
2	Normal	Intermediate State	674	180	1.90	Normal	Normal
3	Normal	Normal	513	140	1.87	Normal	Normal
4	Normal	Normal	447	120	1.89	Normal	Normal
5	Normal	Normal	399	109	1.87	Normal	Normal
6	Normal	Intermediate State	359	106	1.76	Normal	Normal
7	Normal	Normal	305	86	1.82	Normal	Normal
8	Normal	Intermediate State	215	62	1.79	Normal	Normal
9	Normal	Intermediate State	137	45	1.60	Intermediate State	Intermediate State
10	Normal	Normal	81	25	1.69	Critical State	Critical State
11	Arrhythmia	Critical State	1,572	400	1.97	Normal	Normal
12	Arrhythmia	Intermediate State	1,263	340	1.89	Normal	Normal
13	Arrhythmia	Critical State	787	225	1.80	Normal	Normal
14	Arrhythmia	Critical State	606	165	1.87	Normal	Normal
15	Arrhythmia	Intermediate State	562	147	1.93	Normal	Normal
16	Arrhythmia	Critical State	92	25	1.88	Critical State	Critical State
17	Arrhythmia	Critical State	84	30	1.48	Critical State	Critical State
18	Arrhythmia	Critical State	252	64	1.97	Normal	Normal
19	Arrhythmia	Critical State	140	36	1.95	Intermediate State	Intermediate State
20	Arrhythmia	Intermediate State	198	56	1.82	Intermediate State	Normal
21	Arrhythmia	Intermediate State	174	56	1.63	Intermediate State	Normal
22	Arrhythmia	Intermediate State	165	52	1.66	Intermediate State	Normal

It is important to note that, although the methodology had been applied to individuals over 20 years of age until now, the values of the cases between 18 and 20 years included in this study showed similar values to those presented in the rest of the sample, which would suggest that the methodology may be applicable to this age range. However, since it is a very small population, further studies are required to assess its applicability in this population or other demographic groups.

## Results of the statistical analysis with the original limits

According to these mathematical limits, 161 normal cases, 89 in an intermediate state, and 23 in a critical state were found. When comparing these mathematical limits with the conventional diagnosis, the following values were obtained with the original limits: a sensitivity of 0.595, accuracy of 0.636, a specificity of 0.653, a positive predictive value (PPV) of 0.412, a negative predictive value (NPV) of 0.797. The Cohen's Kappa coefficient for the 2 × 2 table was 0.219 (95% CI, 0.104–0.334), and for the 3 × 3 table, it was 0.141 (95% CI, 0.050–0.220). See Table 3.

**Table 3:** Comparison between the original limits and the adjusted limits for the Peruvian population.

Metric	Original (Kp ≤ 200)	Ajustado/Youden (Kp ≤ 144)
TP	47	34
FP	67	31
FN	32	45
TN	126	162
Sensitivity (95% CI)	0.595 (0.481–0.696)	0.430 (0.329–0.544)
Specificity (95% CI)	0.653 (0.591–0.715)	0.839 (0.788–0.886)
PPV (95% CI)	0.412 (0.347–0.478)	0.523 (0.425–0.630)
NPV (95% CI)	0.797 (0.750–0.844)	0.783 (0.750–0.819)
Accuracy (95% CI)	0.636 (0.581–0.691)	0.721 (0.673–0.768)
F1-Score (95% CI)	0.487 (0.410–0.560)	0.472 (0.373–0.570)
Cohen's Kappa (2 × 2, 95% CI)	0.219 (0.104–0.334)	0.285 (0.164–0.409)
Cohen's Kappa (3 × 3, 95% CI)	0.141 (0.05–0.22)	0.123 (CI 0.036–0.209)
AUC (Score-based, 95% CI)	0.596 (0.512–0.678)	0.596 (0.512–0.678)
AUPRC (Score-based, 95% CI)	0.457 (0.372–0.561)	0.457 (0.372–0.561)
Brier Score (Score-based, 95% CI)	0.206 (0.199–0.206)	0.206 (0.199–0.206)
AUDCA (Score-based, 95% CI)	0.045 (0.044–0.052)	0.045 (0.044–0.052)

PPV, positive predictive value; NPV, negative predictive value; CI, confidence interval.

## Results of establishing new ranges according to the characteristics of the Peruvian population sample

After conducting the previous analyses, the ranges were adjusted to improve their accuracy in the Peruvian population. With this adjustment, the classification changed as follows: values between 0 and 99 correspond to a critical state, those between 100 and 144 indicate an intermediate state, while values above 145 are considered normal.

With the new ranges, the following values were found: a sensitivity of 0.430, accuracy of 0.721, a specificity of 0.839, a positive predictive value (PPV) of 0.523, a negative predictive value (NPV) of 0.783. The Cohen's Kappa coefficient for the 2 × 2 table was 0.285 (95% CI, 0.163–0.409), and for the 3 × 3 table, it was 0.123 (95% CI, 0.036–0.209). See Table 3.

With the concordance values found from the adjustment of the limits, it was determined that the accuracy, which refers to the total proportion of correctly classified cases (both true positives and true negatives), achieved a value of 0.721. The improvement in this parameter compared to the original limits, where the accuracy was 0.636, indicates that the method with the adjusted limits is more suitable for the overall classification of cases.

**Figure 3:**
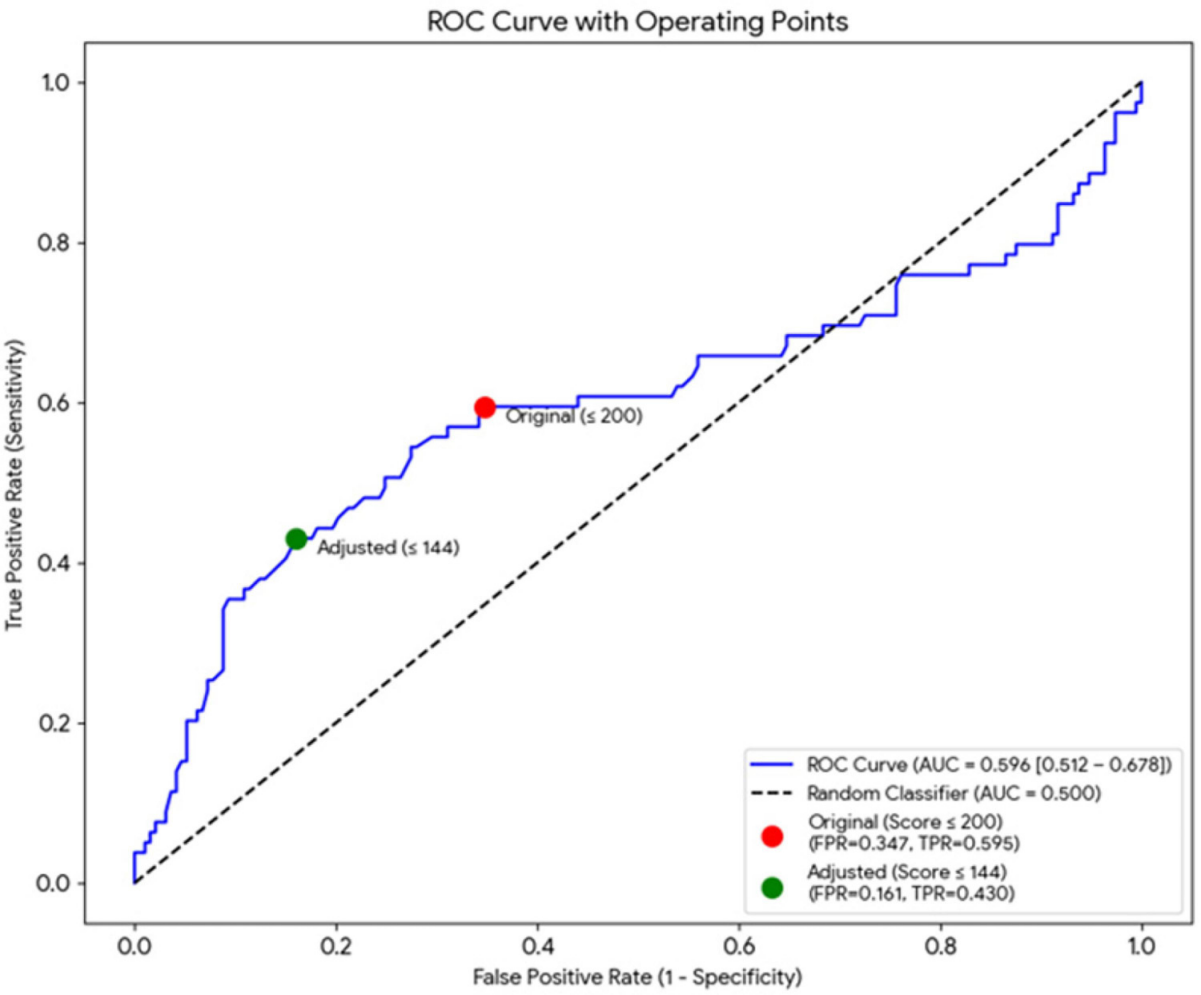
ROC curve for Kp (*n* = 272). The area under the curve (AUC) was 0.596 (95% bootstrap CI 0.498–0.680). The operating points corresponding to the original (Kp ≤ 200) and adjusted/Youden (Kp ≤ 144) thresholds are indicated.

In the full sample (*n* = 272; 79 with arrhythmia), the discrimination of the Kp index was modest: AUC = 0.596 (95% bootstrap CI 0.498–0.680). Using the original binary criterion (Kp ≤ 200 is arrhythmia), the sensitivity was 0.595 (95% CI 0.479–0.704) and the specificity was 0.653 (95% CI 0.581–0.720). After adjusting the limits (Kp ≤ 144, which coincides with the Youden threshold in this sample), the sensitivity fell to 0.430 (95% CI 0.319–0.547) while the specificity increased to 0.839 (95% CI 0.780–0.888). The details (TP/FP/FN/TN and PPV/NPV with their 95% CIs are shown in Table 3 and the ROC curve in Figure 3. In absolute terms, the adjusted threshold increases the proportion of false negatives (45 vs. 32 in the sample) and decreases false positives; for every 1,000 subjects with the prevalence of this sample, the adjustment would imply ∼48 additional undetected arrhythmias but would avoid ∼132 false positives. The estimated distribution of correct and incorrect classifications per 1,000 screened patients is summarized in Table 4, illustrating the clinical impact of the adjusted threshold.

**Table 4:** Distribution of correct and incorrect classifications per 1,000 screened patients.

Clinical Impact Table (per 1,000 Patients)	
Metric	Adjusted (User: Score ≤ 144)
Patients with Arrhythmia (Actual)	290
Healthy Patients (Actual)	710
TP (Correctly Detected)	125
FN (Missed)	165
TN (Correctly Healthy)	596
FP (False Alarms)	114

Additionally, it was found that Cohen's Kappa coefficient for the 2 × 2 classification with the adjusted methodology indicates a fair agreement according to the Landis and Koch scale (0.285), contrasting with the results obtained with the original limits, which yielded a slight value (0.219). For the 3 × 3 table, the Kappa coefficient was 0.123, indicating insignificant agreement, meaning it remained in the same classification regarding the original methodology, according to the mentioned scale. The measures obtained with this methodology using the adjusted limits demonstrate a moderate ability to differentiate between normality and arrhythmia for the Peruvian population, although it is not useful for stratifying the severity level of cardiac dynamics into three levels (normal, intermediate, critical).

## Discussion

Cardiovascular diseases constitute and remain the leading cause of death worldwide, highlighting the importance of continuously developing increasingly accurate methods for diagnosing heart conditions (26). This statement holds true both globally and within the specific context of Peru, where cardiovascular diseases have a significant social and economic impact that tends to increase over time (14, 26). For this reason, over the past 40 years, there has been extensive research aimed at achieving new interpretations of cardiac signals using various mathematical methods, which have yielded significant and promising results (27).

The study of cardiac dynamics through nonlinear dynamic methods, such as Poincaré diagrams, entropy, and symbolic dynamic theories (27, 28), has demonstrated that the conception of normality and disease needs to be reevaluated, along with several concepts that form this understanding. The concept of variability has been reconsidered based on new perspectives that depart from classical concepts like homeostasis, regularity, or periodicity as ideal physiological parameters. Studies have shown that excessive periodicity, as well as highly variable behaviors, are associated with abnormal states or those related to cardiac pathologies, while normal states are found in intermediate points—neither too variable nor too periodic—thus contradicting the classical concept of homeostasis (29). Additionally, recent studies have found that it is crucial to conduct prolonged monitoring in high-risk patients, as many episodes of cardiac pathologies go undetected during short 24-hour monitoring periods (30). Consequently, there is a suggestion to combine mathematical approaches with other more detailed diagnostic methods to optimize the detection of cardiac pathologies.

Although different applications of fractal geometry in medicine have been developed (31, 32) to characterize normality and disease, most of these studies are based on the comparison of isolated fractal measures and the search for characteristic value ranges of normality or disease, which have not proven definitive for establishing distinctions. Based on the comparison of fractal dimensions, it is not possible to establish a differentiation between normality and disease in cardiac dynamics, as shown in other studies (30). Rather, it is the occupation spaces that may enable a differentiated evaluation in certain specific cases.

Following this line of research, the methodology applied in this study emphasizes the importance of using appropriate methods to address the chaotic and irregular nature of the heart rhythm, in order to establish precise, objective, and reproducible measures of its dynamics. However, the concordance study results relative to the gold standard show much more modest values than those reported in previous studies (21, 22, 33), even taking into account the adjustment of the limits, indicating that the method is relatively sensitive but not very specific, which may be due to previous methodological errors or differences in the evaluated samples. Observing the kappa coefficient of 0.29 obtained in the 2 × 2 table, it is evident that the method only has fair agreement with the gold standard, according to the Landis and Koch index. Furthermore, the agreement is even lower when evaluating the stratification capacity with the 3 × 3 table, which obtained a kappa coefficient of 0.13, equivalent to slight agreement. The results cast doubt on the method's ability to stratify the severity of cardiac dynamics. In this regard, due to the small sample size in this study, it is important to conduct further research to establish the applicability of the limits defined in this study to the rest of the Peruvian population or to generate additional adjustments according to their characteristics. Similarly, it is important to apply the methodology in other specific populations to verify its diagnostic capacity before any clinical application.

The technique based exclusively on the Kp index does not demonstrate sufficient discriminatory capacity for use as a sole screening method, given that the area under the curve (AUC) obtained is low and the sensitivity is inadequate at the threshold that minimizes false positives (34, 35). The adjusted threshold (Kp ≤ 144) optimizes overall accuracy and specificity in the analyzed sample (according to the Youden criterion), although it does so at the expense of a significant loss of sensitivity (36).

In this context, if the purpose of the method is the detection or screening of arrhythmias, it is not recommended to use the adjusted threshold as the sole test to rule out abnormalities (37). Instead, it would be preferable to implement a two-stage diagnostic strategy, initially using a more sensitive threshold that allows for preliminary detection, followed by a second confirmation phase through manual review, extended recording or full Holter monitoring (38). Alternatively, the algorithm could be retrained or extended by incorporating additional parameters—such as the Kg index, spectral density (FD) factor, temporal variables, and relevant clinical data—and subsequently subjected to an external validation process (25, 37, 39). On the other hand, if the primary objective is to reduce the false positive rate in a clinical setting where diagnostic confirmation is not an operational problem, the use of the adjusted threshold could be considered justifiable, provided the inherent limitations of this approach are explicitly acknowledged (40, 41).

In anticipation of future research and consistent with the current line of work, it is considered pertinent to delve deeper into several analytical fronts. First, a stratified subanalysis by type of arrhythmia (e.g., atrial fibrillation, extrasystoles, or ventricular tachycardia) is of interest, given that the methodology could exhibit differential discriminatory performance depending on the nature of the rhythm disorder (41). It would also be relevant to perform an analysis by age and sex, aimed at the specific calibration of thresholds, as well as to evaluate the potential influence of artifacts on model performance metrics (39, 40).

Finally, the development of nonlinear combined models (such as decision trees, ensemble methods, or the inclusion of new time variables) could be explored, always within a rigorous methodological framework that includes internal cross-validation and evaluation in external samples, to prevent overfitting and preserve the external validity of the findings (37).

In conclusion, we could argue that the severity of a false negative depends on the type of arrhythmia: for example, failure to detect atrial fibrillation (AF) can delay the initiation of anticoagulation and prevent stroke; Failure to detect sustained ventricular tachycardia can delay urgent evaluation and treatment.

Given the high percentage of undetected arrhythmias (40%–57% depending on the threshold), routine use of this method as a single, definitive screening test to detect all arrhythmias in clinical practice is not recommended if the ultimate goal is to capture the highest possible proportion of events (screening). Therefore, everything must be correlated with clinical judgment. However, if the proposed application is filtering to reduce false positives (for example, a detector used to reduce unnecessary Holter readings), then the adjusted threshold (Kp ≤ 144) may make sense, always keeping in mind that the test is not sensitive enough to rule out arrhythmia (high NPV but low sensitivity) and therefore does not replace absolute clinical judgment (40).

## Data Availability

The data analyzed in this study is subject to the following licenses/restrictions: In accordance with the ethical standards outlined in the Código de Ética para la Investigación y la Integridad Científica (CE-VRINV-001/V03) of the Universidad Católica de Santa María, this study adheres to the principles of beneficence, non-maleficence, justice, and autonomy. The research has been classified as minimal risk, as it involves the analysis of non-invasive medical data from clinically prescribed examinations. The study ensures the protection of participant confidentiality and privacy, as mandated by the Resolución Ministerial N° 233-2020-MINSA regarding ethical considerations for research involving human subjects. All sensitive data has been anonymized to safeguard participant identity. Requests to access these datasets should be directed to Sandra Catalina Correa scatalinacorreah@gmail.com.
